# Improved computational method to generate properly equilibrated atomistic microstructures

**DOI:** 10.1016/j.mex.2021.101217

**Published:** 2021-01-06

**Authors:** Ankit Gupta, Satish S. Rajaram, Gregory B. Thompson, Garritt J. Tucker

**Affiliations:** a Department of Mechanical Engineering, Colorado School of Mines, Golden, CO 80401, USA; b Department of Mechanical Engineering, Drexel University, Philadelphia, PA 19104, USA; c Department of Metallurgical and Materials Engineering, University of Alabama, Tuscaloosa, AL 35487, USA

**Keywords:** Thermal equilibration, Atomistic simulations, Nanocrystalline structure

## Abstract

Atomistic simulations play an important role in unravelling the fundamental behavior of nanocrystalline (NC) metals/alloys. To ensure the validity of the simulated results, the initial NC structures must be representative of a real material to the extent possible. Using proper equilibration techniques, it must also be ensured that these NC structures reach a state of metastable equilibrium before probing their response. To this effect, the influence of simulated thermal equilibration of atomistic NC Ni structures on the resulting mechanical behavior is discussed in this work. It is shown that the well-equilibrated NC structures become stiffer in terms of both elastic response and yielding behavior and accumulate less residual strain upon unloading, thus, signifying the importance of proper equilibration. However, it is found that the regular equilibration method of thermal relaxations at 300 K, typically employed in atomistic modeling studies, takes significantly longer to drive the NC structures towards a metastable equilibrium state. Finally, an improved two-step equilibration method is presented that drastically expedites the equilibration process while resulting in the structural and mechanical properties comparable with the regular equilibration method performed for significantly larger simulation times. The major modification in the improved method involves:

• *Subjecting only the grain boundary and the surrounding atoms to thermal relaxations at relatively higher temperature*.

## Graphical abstract

**Figure:**
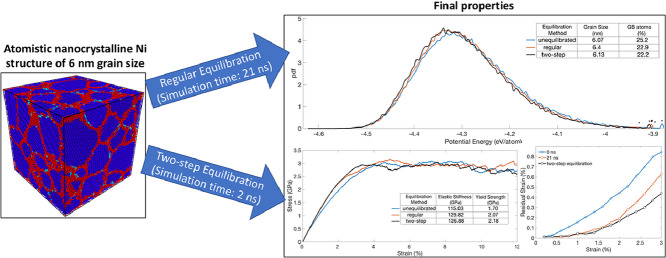

**Specifications table**

**Table:** 

Subject Area:	Materials Science
More specific subject area:	*Computational Materials Science*
Method name:	*Improved Equilibration Method for Atomistic Microstructures*
Name and reference of original method:	*Regular thermal equilibration of atomistic microstructures at 300 K* • Van Swygenhoven, et al. “*Plastic behavior of nanophase Ni: A molecular dynamics computer simulation*.” APL (1997). • Van Swygenhoven et al. “*Characterisation of the microstructure of nanophase Ni: a molecular dynamics simulation study*.” Nano Mat. (1999). • Van Swygenhoven et al. “*Grain-boundary structures in polycrystalline metals at the nanoscale*.” Phy Rev B (2000). • Schiøtz et al. “*Atomic-scale simulations of the mechanical deformation of nanocrystalline metals*.” Phy Rev B (1999). • J. Gruber et al. “*Development of physically based atomistic microstructures: The effect on the mechanical response of polycrystals.*” Comp. Mater. Sci. (2017).

## Method details

Nanocrystalline (NC) materials have attracted widespread attention from the scientific and engineering communities in the past few decades, due to several unique properties such as greater strength and ductility, higher fatigue resistance and radiation tolerance, as compared to their coarse-grained counterparts [1]. It is noted that the origins of potential advancements in these functional properties lie in the larger volume fraction of interfaces between grains (i.e., grain boundaries (GBs)) present in NC materials - as the average grain-size decreases, the fraction of GBs within the microstructure correspondingly increases [1]. As such, several studies have focused on probing the role of interfaces in dictating superior behavior of NC materials using computational modeling/simulation approaches. Unlike experimental studies, it is easier to isolate and study the behavior of interfaces at the nanoscale in computational studies employing atomistic simulation techniques, as full atomic resolution is attainable throughout the simulation process [2]. One critical consideration in atomistic simulations is the initial NC structure that is used to model the desired behavior. In order to capture the correct physical behavior, the initial atomistic microstructure must be as closely representative of a real material as possible in terms of grain size, shape distributions and interfacial character. There exist several techniques in literature related to the generation of NC microstructures that are employed in atomistic simulations. Some more commonly used methods, such as Voronoi tessellation, result in unrealistic NC structures with artifacts like improper shapes/structures of grains, interfaces and triple junctions [3]. Since, properties of NC materials are vastly affected by their interface character, it is vital to ensure that the initial NC structures used in atomistic simulations studies are devoid of any such artifact that can significantly bias their outcome. Subsequently, several continuum scale simulation approaches, based on phase field [4], front-tracking/vertex [5], and Monte Carlo Potts modeling techniques [6], have been employed to generate more physical NC structures having curved GBs, improved triple junction angles and grain size distributions that are closer to experimental measurements. These more physically based microstructures, after proper post-processing, can be used for atomistic simulations, as recently demonstrated by Gruber et al., to be having substantially different strain accommodation behavior of deformation mechanisms, as compared to Voronoi microstructures [4].

When employing atomistic modeling methods to probe the response of NC materials, it is also paramount to ensure that the initial microstructures are in a state of metastable equilibrium. Otherwise, the resulting trends/data will likely suffer inaccuracies from unrealistic starting structures biasing the operative nanoscale mechanism(s) that govern materials response during simulations. The term ‘metastable’ is used in this case since a perfectly ‘stable’ equilibrium state for a pure NC material is theoretically a single crystal, lacking the presence of all interfaces, such as GBs [7]. In the existing literature, the discussion related to the proper equilibration techniques of as-generated atomistic microstructures is limited, and details vary significantly. While subjecting the atomistic microstructures to thermal relaxations at 300 K is the general equilibration technique used in many studies [8], [9], [10], [11], [12], [13], [14], the reported duration of such molecular dynamics (MD) simulations can vary from as low as 20 ps [8,9] to a few nanoseconds [10,14]. In some studies [7,^14^,15], thermal relaxations were conducted at higher temperatures (400–600 K) to expedite the equilibration process. However, equilibration at high temperatures for time periods longer than what were typically used in such studies (10–50 ps), could lead to substantially different microstructures originating from grain-growth in NC materials. There are even fewer studies discussing the influence of the amount/nature of thermal equilibration on properties of NC materials. In [14], Schiøtz et al. reported that the unequilibrated microstructures generated from Voronoi Tessellation are generally softer than the equilibrated ones because of non-equilibrium atomic structure of the GBs in higher energy states – supporting the need for such investigation and improved equilibration methods. It should also be noted here that apart from the macroscopic and microscopic degree of freedoms, GB energy depends on the defect density, and generally, it is varied in atomistic simulations either directly [16] or by using non-periodic simulation cells, where, open surfaces can act as defects source/sink [17]. However, GBs in NC metals have been shown to contain more disorder or excess free volume than their equilibrium counterparts with same five degrees of freedom that results in higher energy, thus, making non-equilibrium GBs and triple junctions as efficient sinks for defects [18].

In this work, we study the effect of thermal equilibration of atomistic NC Ni structures on the resulting properties, using regular equilibration method, i.e, thermal relaxations at 300 K, which is typically employed in MD studies. Specifically, we focus on the mechanical properties of the NC structures. It is shown that while proper equilibration is important, the regular equilibration method takes substantially longer to drive the NC structures towards a metastable equilibrium state. An improved two-step equilibration method is then presented. This method allows subjecting the NC structures to thermal relaxations at higher relative temperatures while purposely restricting any major grain growth in the resulting microstructure, thus significantly expediting the equilibration process and saving computational resources. The parameters of the two-step equilibration method are further varied and validated to ensure that the resulting NC structures and the associated mechanical properties are comparable to the regular equilibration method performed for significantly larger simulation times.

### Regular equilibration method- thermal relaxations at 300 K

**Fig. 1:**
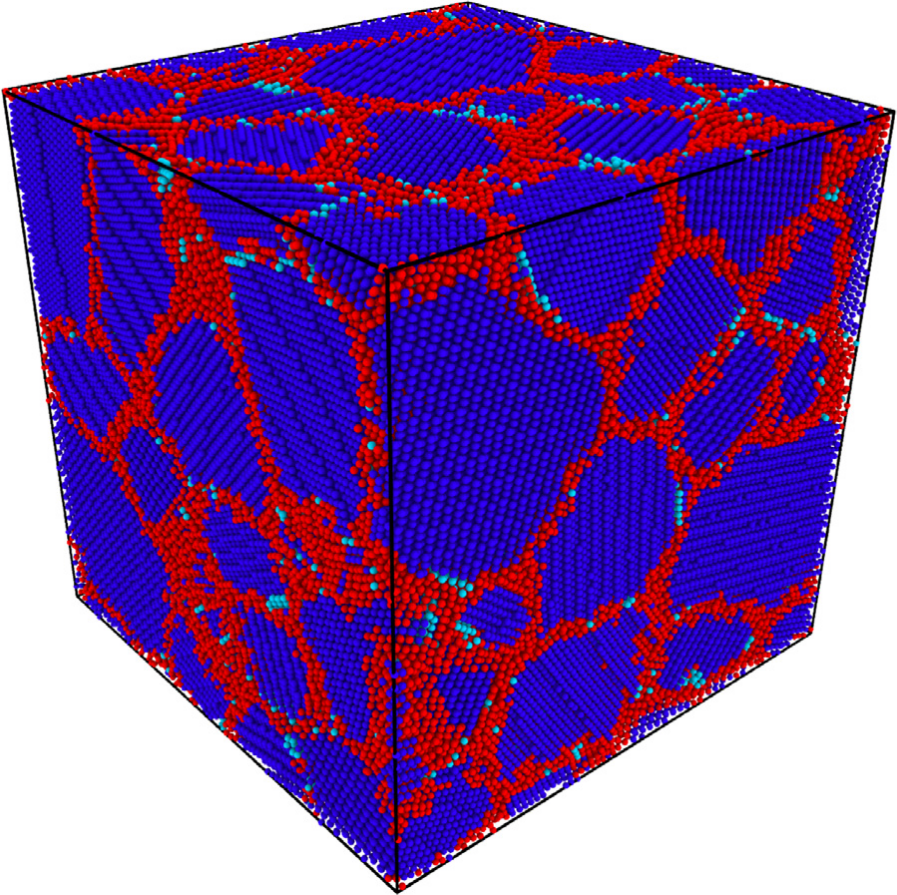
A 6 nm grain size phase field generated atomistic Ni NC structure. (blue atoms: FCC, cyan atoms: HCP, red atoms: GB).

Initial atomistic NC structures employed in our simulations were generated from a phase-field based approach, as outlined by Gruber et. al [4]. Since curved GBs present in phase-field generated microstructures are more prone to atomic rearrangements, studying the role of proper equilibration in such microstructures becomes critically important. The initial NC structure (shown in Fig. 1) had an average grain size of 6 nm, a random texture and periodic boundaries in each dimension. The dimensions of the simulation box were 18 nm × 18 nm × 18 nm. It contained around 40 grains, and 500,000 atoms. All simulations were performed using the LAMMPS package [19] employing an Embedded Atom Model (EAM) interatomic potential for Ni [20]. The visualizations were done in OVITO [21]. The average grain size and the total number of grains were determined by characterizing the atomistic microstructure using the method outlined in [22]. We employed the regular equilibration method that is commonly used in literature [12], [13], [14], to bring this initial NC structure to a metastable equilibrium state. Using MD simulations, we subjected the initial NC structure to thermal relaxations at 300 K in an isothermal-isobaric (i.e., NPT) ensemble while maintaining zero normal pressure at all periodic boundaries. A time-step of 1 fs was chosen for this simulation.

**Fig. 2:**
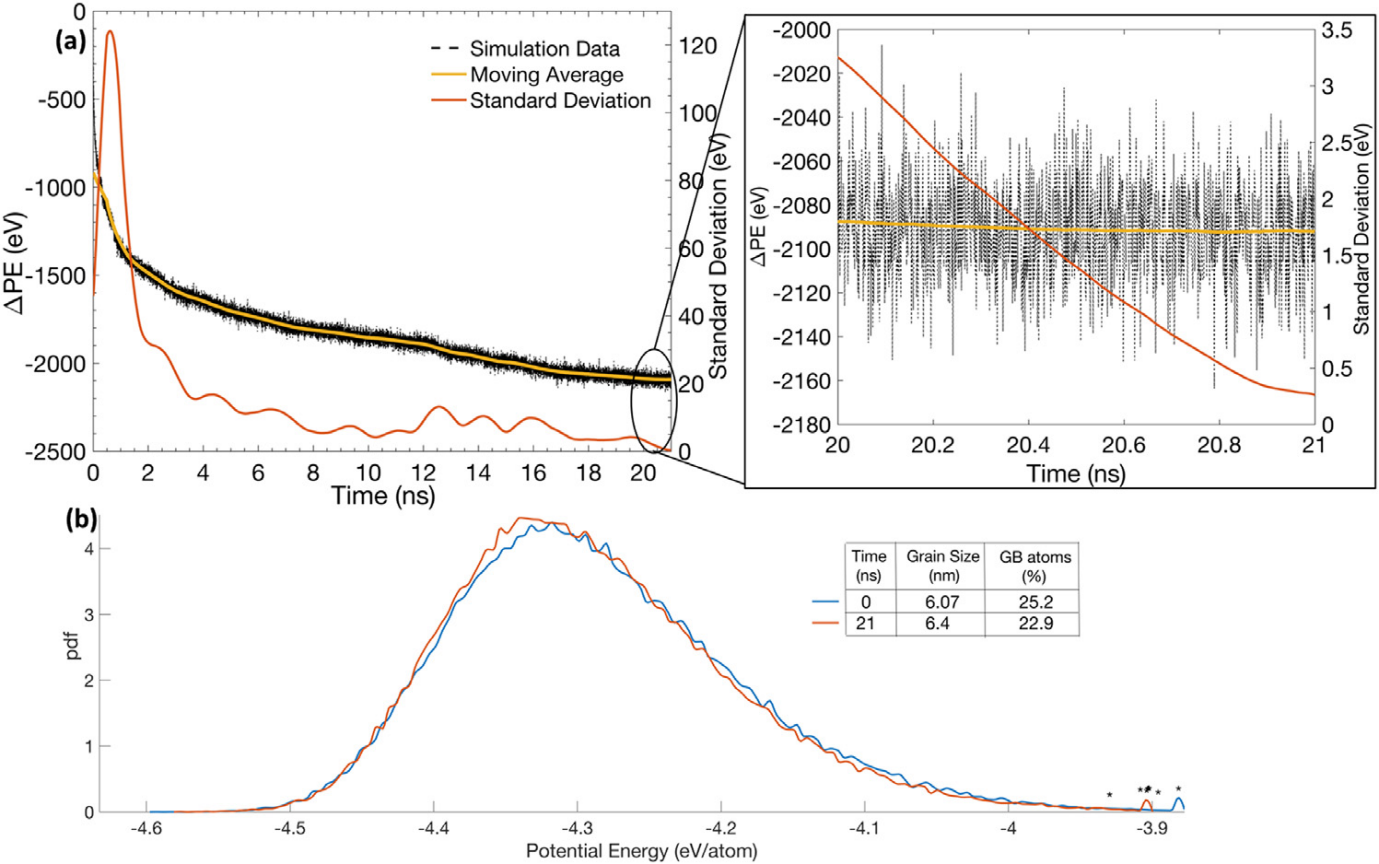
(a) Evolution of system's potential energy with respect to the initial unequilibrated configuration, as a function of simulation time, during the regular equilibration of 6 nm grain size NC structure (inset shows variation during the last nanosecond). (b) Microstructure statistics and GB atomic potential energy distributions at the beginning and end of simulation.

Fig. 2a shows the evolution of the system's potential energy with respect to the initial unequilibrated configuration, as a function of simulation time, during the regular 300 K thermal equilibration of the 6 nm average grain size NC Ni structure. It can be observed from this figure that the potential energy (black curve) initially decreases rapidly and seems to become relatively constant during the last nanosecond of the simulation, i.e., 20–21 ns, (as shown in the inset to the figure). To remove small fluctuations in the potential energy data, the moving average of the potential energy is plotted as a function of time. The plot (yellow curve) also appears to plateau during the last nanosecond of the simulation. Therefore, it can be qualitatively established that the NC structure is now more properly equilibrated, as compared to the configurations at shorter equilibration times (e.g., 20-1000 ps) that are often used in MD studies. Thus, the simulation can be stopped at 21 ns. To properly quantify the variation of potential energy, the standard deviation of the moving average data over a moving window of 1 ns was calculated and plotted as a function of simulation time (orange curve). It can be observed from Fig. 1a (inset), that the standard deviation averages at 1.5 eV during the last nanosecond and falls below 0.5 eV at the end of simulation (i.e., 21 ns). Such a small value of standard deviation indicates that the system's energy has become relatively constant after 21 ns, and thus, the system is now properly equilibrated. It should be noted here that the equilibration time will increase if either the system size increases, or the average grain size and the equilibration temperature decreases.

Fig. 2b shows some microstructure statistics at the beginning and the end of the equilibration simulation. There is no significant change in average grain size of the microstructure during equilibration. It should be noted here that all atoms in the system do not relax by the same amount. The decrease in the total potential energy (~2100 eV) mostly happens due to a reduction in the fraction of GB atoms from 25.2% to 22.9%, which accounts for 88% of the total energy change. Rest of the reduction in the system's potential energy occurs as the NC GBs structures become relatively more ordered at the end of simulation. However, this contribution is small as the potential energy distribution plots of GB atoms in the two configurations appear nearly identical but with minor details such as the average energy of GB atoms decreases from -4.28 eV/atom to -4.29 eV/atom in the equilibrated configuration. Interestingly, the average energy of grain FCC atoms remains constant at -4.399 eV/atom during equilibration. This value is close to the cohesive energy of Ni atom calculated from the selected EAM potential, -4.45 eV/atom [20], after accounting for thermal contributions at 300 K (~0.04 eV/atom). It indicates that the grain FCC atoms are already near their equilibrium state and, hence, their contribution to the total energy reduction should be small despite their large volume fraction.

In order to study the effect of thermal equilibration on mechanical behavior, NC structural configurations, corresponding to various time-steps during thermal equilibration, are subjected to uniaxial tensile loading simulations. Using MD, a constant strain-rate of 10^8^ s^−1^ is applied along one direction of the simulation cell while maintaining zero normal pressure at periodic boundaries along the transverse directions. These simulations were performed at 300 K in an NPT ensemble. The structures were loaded to a total uniaxial tensile strain of 12%. The normal stress along the loading direction is plotted as a function of applied strain. While there are several ways to calculate stress in a MD simulation [23], in this case, the global pressure tensor of the system is used. In order to reduce the effect of thermal noise, the average stress data is obtained at every 1000 time-steps as an average of the last 100 time-steps. The initial elastic stiffness is calculated as the slope of the stress-strain curve below 1% strain. The yield strength is calculated using the 0.2% offset method [24]. Additionally, in order to study the effect of thermal equilibration on yielding behavior of these NC structures, the microstructure configurations (atomic positions and velocities) at every 0.2% strain increments, till a total loading strain of 3% (> 0.2% offset yield point), are stored in restart files, thus enabling a simulation restart from any given point. These configurations are then unloaded to a state of zero normal stress by applying a constant but equal strain-rate in the direction opposite to initial loading. The resulting residual strain in the system along the loading direction is then calculated to probe yielding behavior. More details regarding the calculation of residual strain can be found in [24,25].

**Fig. 3:**
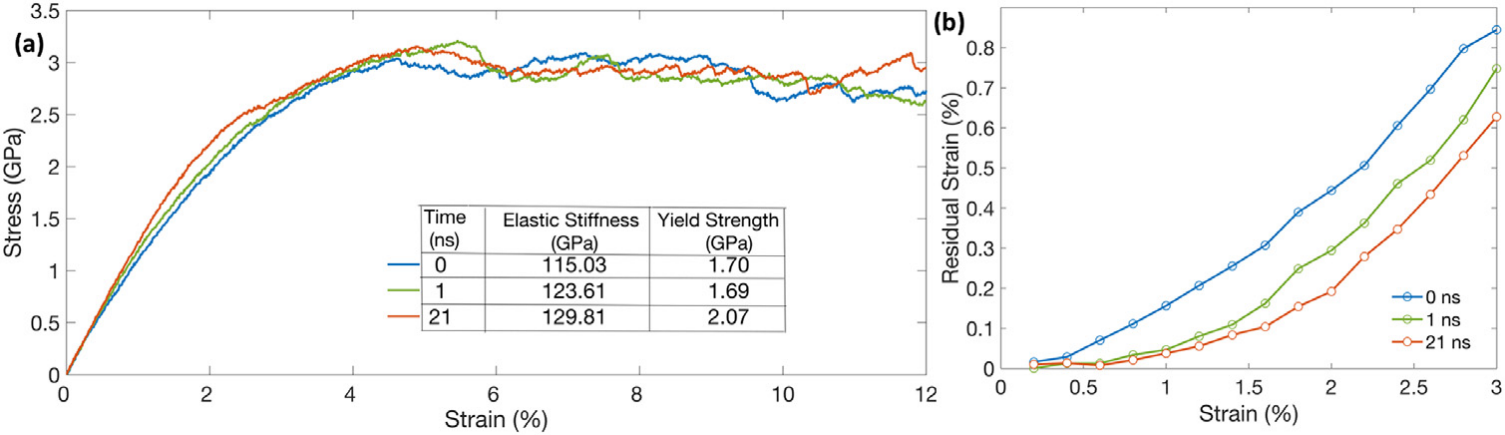
(a) Stress-strain curves obtained from the uniaxial testing and (b) residual strain curves obtained from the unloading simulations of 6 nm grain size NC structure at various time-steps during the regular equilibration.

Fig. 3(a) and (b) show the stress-strain and residual strain curves obtained from the uniaxial tensile loading and unloading simulations of the NC structures, at various time-steps during the regular thermal equilibration simulation. The initial elastic stiffness and the yield strength values are also shown in Fig. 3(a). It can be observed from this figure that the NC structure becomes stiffer in terms of both the initial elastic and the yielding response, i.e, the stiffness and the yield strength values increase as the structure is properly equilibrated, from 115 to 130 GPa and 1.7 to 2.07 GPa, respectively. The higher yield strength of the equilibrated NC structure further indicates a lesser deviation in the material's response from the initial elastic behavior due to activation of fewer microplasticity events at the GBs [24]. It is also reflected in lower residual strain values in the properly equilibrated NC structure prior to macroscopic yielding, as seen in Fig. 3(b), indicating accumulation of small plastic strain. These data together suggest that the amount/time of thermal equilibration can significantly affect the mechanical response of a material, specially at smaller grain sizes. It is therefore important to ensure that the NC structures, used in atomistic modeling studies, are properly equilibrated. However, in the present case of 6 nm NC structure, it is found that the regular equilibration carried at 300 K, takes approximately 21 ns till a metastable equilibrium state is reached. Given the timestep size of 1 fs commonly employed in MD simulations of metallic materials, this translates to at least 21 million time-steps, which makes regular thermal equilibration method computationally expensive. Therefore, the need of expediting the equilibration process from a computational perspective becomes critical. As such, our improved two-step equilibration method is presented next, and the results are compared with the regular thermal equilibration method.

### Two-step equilibration method

To expedite the equilibration process, it is generally carried out at higher temperatures (400–600 K) in previous atomistic studies [7,^14^,15]. In these studies, the annealing durations were very small (10–50 ps). Consequentially, no significant grain growth was reported. However, imposing higher temperatures during thermal equilibration could lead to significant grain growth, especially in NC materials which have larger fraction of higher energy interfacial structures. It results in a larger thermodynamic driving force for grain growth in NC systems to minimize the total energy by reducing interfacial area. For instance, the average grain size of the NC Ni structure, employed in this study, grew from 6 nm to 7.3 nm and 10.5 nm at annealing temperatures of 800 K and 1000 K, respectively, only after 1 ns. One way to prevent grain growth might be subjecting only the GB and the surrounding edge atoms to high temperature equilibration and fixing the remaining grain center atoms. Moreover, as shown earlier, it is only the GB atoms that contributed the most towards the decrease in the system's potential energy during regular thermal equilibration simulation. Thus, we propose a two-step equilibration method.

**Fig. 4:**
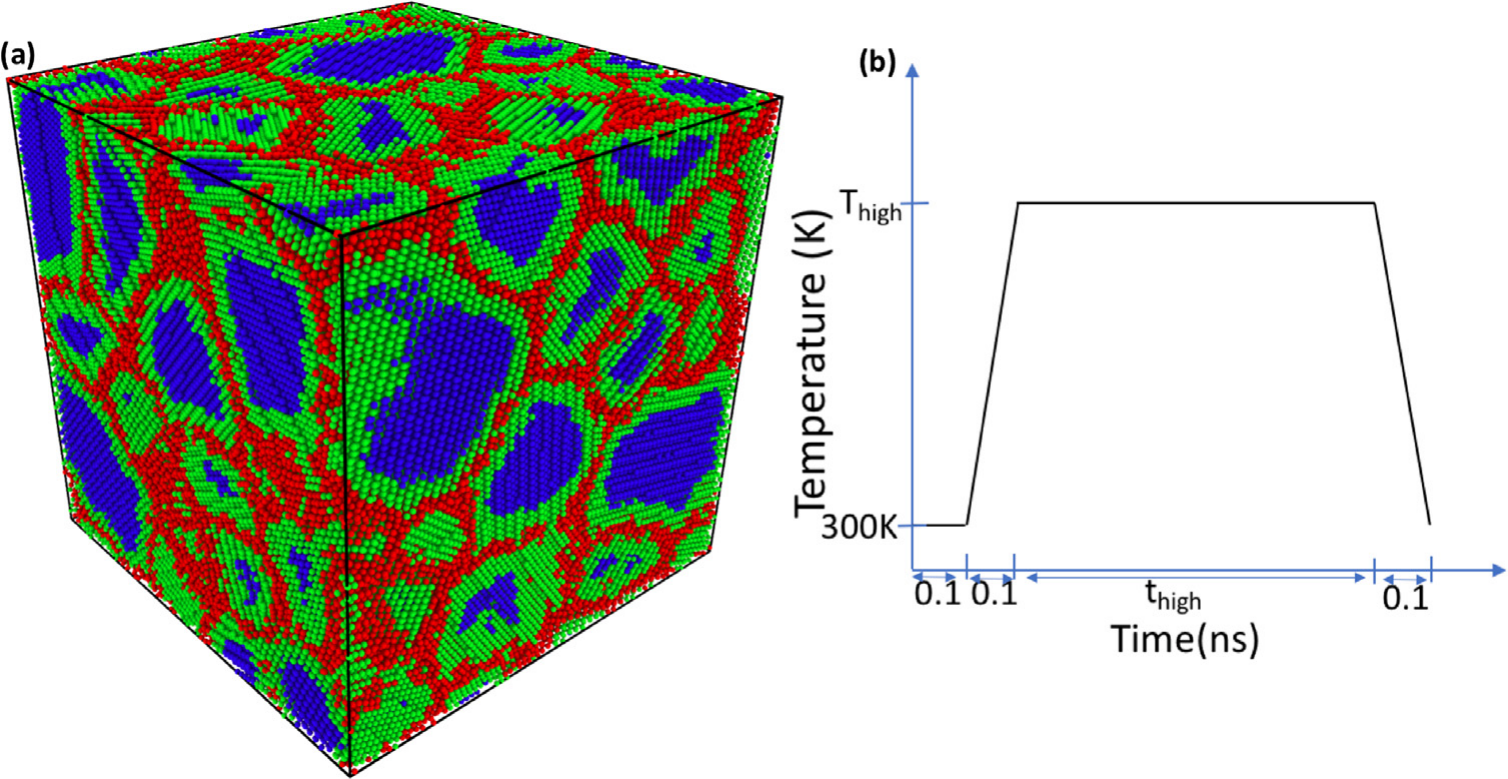
(a) Delineation within the atoms of the initial NC structure: Blue: grain center atoms, Green: grain edge atoms, and Red: GB atoms (b) Temperature profile of the grain edge and the GB atoms during the first step of the two-step equilibration.

In the first step, only the GB and the grain edge atoms were equilibrated at high temperature and the positions of the grain center atoms were fixed. This delineation within the atoms of the NC structure is labeled and shown accordingly in Fig. 4(a), motivated by a procedure previously used in tracking the evolution and growth of individual grains in NC Ni [22,26]. The grain center atoms, shown in blue, are atoms which have face-centered cubic (FCC) structure along with every neighboring atom within six nearest neighbor shells. In Ni, this distance is roughly 6.1 Å., which corresponds to the minimum distance required to distinguish atoms of two neighboring grains separated by a low angle grain boundary with a misorientation angle as small as 8.5 degrees. This number was determined by comparing the misorientation angle distribution plots of the grain boundaries in the atomistic nanocrystalline structures with random textures (as obtained from the characterization code) with the Mackenzie Distribution [27]. It should be noted here that this threshold for distinguishing two grains can be further decreased below 8.5 degrees by increasing the neighbor list cutoff beyond sixth neighboring shell. However, the computational cost increases substantially in doing so, while the frequency of boundaries with such small misorientation angles remain low in a randomly textured microstructure [27].

The grain edge atoms, shown in green, are atoms with the FCC structure but not all their neighbors within that cutoff radius are necessarily FCC. The GB atoms, shown in red, lie in a more disordered state and do not have the FCC structure. The high temperature equilibration step was carried out in a canonical (NVT) ensemble, keeping the system's volume fixed. The temperature profile during the equilibration in the first step is shown in Fig. 4(b). The grain edge and the GB atoms were first equilibrated at 300 K for 0.1 ns, followed by heating to some relatively high temperature, *T_high_,* and holding for *t_high_* ns, and finally cooling to 300 K. In order to remove any internal stresses resulting from the constraint on the grain center atoms in the first step, the whole system (i.e., all atoms are mobile) is then subjected to regular thermal equilibration at 300 K in the second step. This step is carried out in an NPT ensemble while maintaining zero normal pressure at the periodic boundaries in every dimension. The results of the two-step equilibration method, as a function of annealing parameters, *T_high_* and *t_high_*, are now discussed.

### *Effect of annealing parameters, T_high_* and *t_high_*

**Fig. 5:**
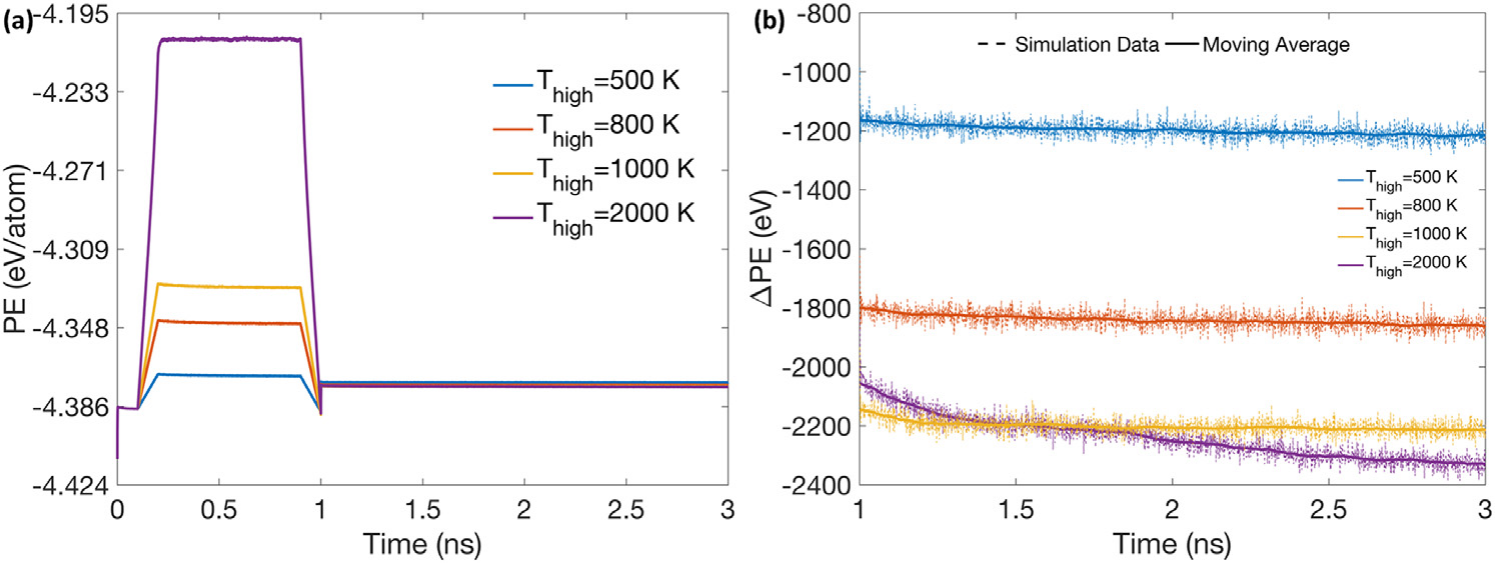
(a) Evolution of total potential energy of the system with time during the two-step equilibration of the 6 nm grain size NC structure, performed at different annealing temperatures *T_high_*. (b) Evolution of total potential energy with respect to the initial unequilibrated configuration, as a function of simulation time, during the second step of the two-step equilibration performed at different annealing temperatures *T_high_*.

Fig. 5(a) reveals the evolution of the system's potential energy with time, during the two-step equilibration of the 6 nm average grain size NC Ni structure (illustrated in Fig. 1), performed at different annealing temperatures *T_high_*. The annealing time *t_high_* was fixed as 0.7 ns in each of these simulations. During the first step of the equilibration process (i.e., time < 1 ns in Fig. 5(a)), the variation of potential energy with time is similar to the temperature variation profile shown in Fig. 4(b), i.e, initially increasing during the heating period followed by a constant value during the high temperature annealing period and finally decreasing as a result of cooling. The constant value of potential energy during the high temperature annealing period depends upon the annealing temperature *T_high_*. In Fig. 5(a), a small dip can be seen in total potential energy at the end of first step before it returns to a higher value at the commencement of the second step, as a result of addition of thermal energy to the previously fixed grain center atoms. During the second step of the equilibration process (i.e., time > 1 ns in Fig. 5(a)), the evolution of the system's potential energy with respect to the initial unequilibrated configuration is more clearly shown in Fig. 5(b). It can be observed from this data that the system's potential energy initially decreases slightly but becomes constant after 1 ns of equilibrating the entire system at 300 K in the second step, (i.e., 2 ns total simulation time), for all cases except when *T_high_* was 2000 K. The value at which the system's potential energy finally stabilizes also depends upon the annealing temperature *T_high_*.

**Fig. 6:**
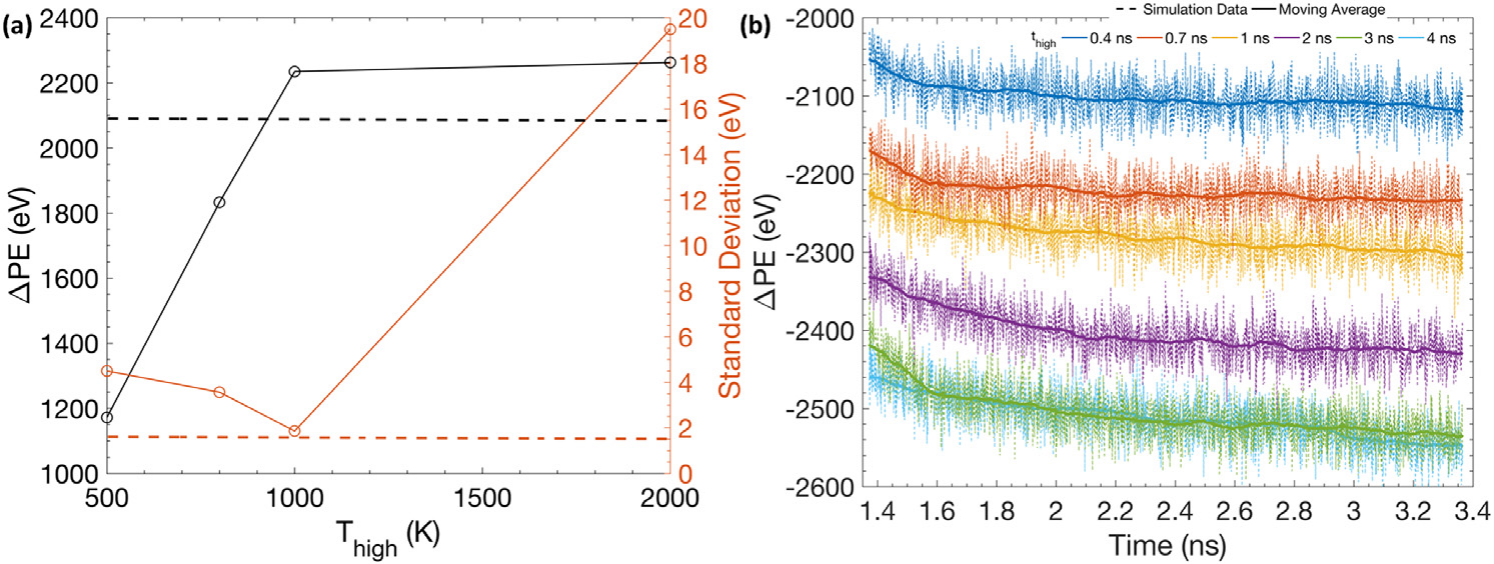
(a) Total decrease in the system's potential energy *∆PE* and the standard deviation of the potential energy data after 2 ns of the two-step equilibration, plotted as a function of annealing temperature *T_high_*. (b) Evolution of total potential energy with respect to the initial unequilibrated configuration, as a function of simulation time, during the second step of the two-step equilibration performed for different annealing times *t_high_*.

Fig. 6(a) shows the total decrease in the system's potential energy (ΔPE) and the standard deviation of the potential energy data after 2 ns of the two-step equilibration, plotted as a function of *T_high_*. The dashed line represents the total potential energy decrease and the standard deviation during the last nanosecond (i.e., 20–21 ns) of the regular thermal equilibration performed earlier at 300 K. ΔPE increases with *T_high_* until 1000 K after which it becomes relatively constant to a magnitude that is slightly higher (~100 eV) than the value obtained in case of regular thermal equilibration performed for significantly longer simulation times. However, the standard deviation is significantly higher when T_high_ was 2000 K as compared to the values at rest of the annealing temperatures. It indicates that the system's potential energy has not yet become constant in this case. Annealing at temperatures higher than the melting point of Ni (T*_m_* ~1600 K) in the first step, might cause large structural rearrangements of the grain edge and the GB atoms, which also continue during the 300 K equilibration in the second step resulting in the further gradual decrease in the system's potential energy during this step, as seen in the case when *T_high_* was 2000 K. Only, annealing performed at 1000 K results in the energetic reduction and the standard deviation that is closest to the regular equilibration case. Therefore, *T_high_* = 1000 K, which lies in between the melting (T*_m_*) and the recrystallization (0.5 T*_m_*) temperature of Ni, is selected as a suitable annealing temperature during the first equilibration step of this proposed two-step equilibration method; since it ensures that atomic relaxations in the first step result in an slightly higher energetic reduction than the regular equilibration case, but also without melting the structure, thereby, allowing the system's energy to stabilize early during the second step.

Although the potential energy change during the two-step equilibration method is sensitive to the annealing temperature *T_high_*, it is not the case with annealing time *t_high_*. Fig. 6(b) shows the evolution of the total potential energy of the system with respect to the initial unequilibrated configuration, as a function of simulation time, specifically during the second step of the two-step equilibration simulations performed for different *t_high_*. The annealing temperature *T_high_* was fixed as 1000 K in these simulations. Little difference is observed between the final constant values of potential energy between the cases investigated (~500 eV total difference between *t_high_* of 0.4 ns and 4 ns). Therefore, in order to save computation time, a smaller value of *t_high_* = 0.7 ns is selected as a suitable annealing time during the first equilibration step. Importantly, the two-step equilibration method, with optimal parameters (*T_high_* = 1000 K and *t_high_* = 0.7 ns), is thereby shown to significantly reduce the equilibration time from 21 ns for regular 300 K equilibration to 2 ns, while resulting in a slightly higher energetic reduction of the simulated atomistic structure. The NC structure, from the two-step equilibration method, and its mechanical properties are next compared to the microstructure obtained from the regular equilibration method after 21 ns, to ensure that high temperature annealing does not lead to significant structural/property changes or any artifacts in the final microstructure.

### Comparison with the regular equilibration method

**Fig. 7:**
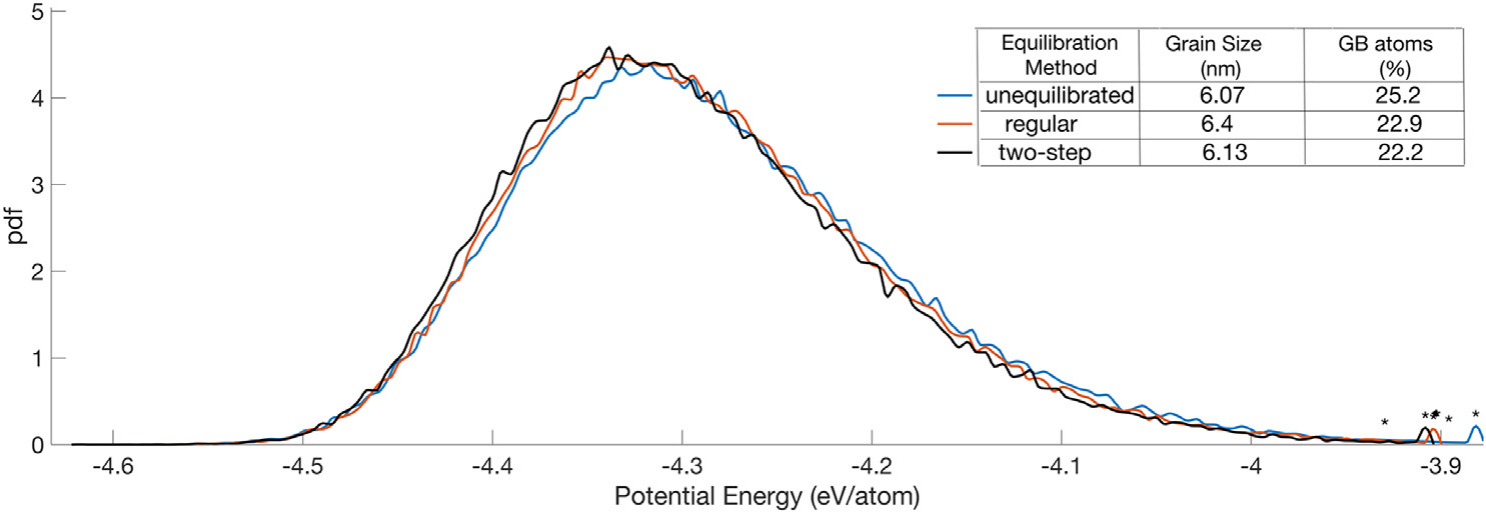
Microstructure statistics and potential energy distribution plots of the GB atoms of the NC structure in the initial unequilibrated and the final equilibrated configurations, as obtained from the regular and the two-step equilibration methods.

Microstructure statistics of the final equilibrated NC structures, as obtained from the two-step and the regular equilibration methods, are shown in Fig. 7. The initial NC structure's statistics are also included. The results show that despite the high temperature annealing performed in the two-step method, minimal grain growth occurs in the resulting structure, comparable to the regular equilibration method. The decrease in the percentage of GB atoms after equilibration is slightly larger in the two-step method, which then also explains the higher energetic reduction observed in this case. Importantly, the potential energy distributions of the GB atoms in the equilibrated NC structures (shown as plots in Fig. 7), as obtained from the two methods, are statistically identical, which indicates to some extent the same degree of disorder in the resulting GB structures in the two cases. The two-step equilibrated NC structure is also subjected to uniaxial tensile loading and unloading simulations, using the methodology discussed earlier, to study its mechanical behavior.

**Fig. 8:**
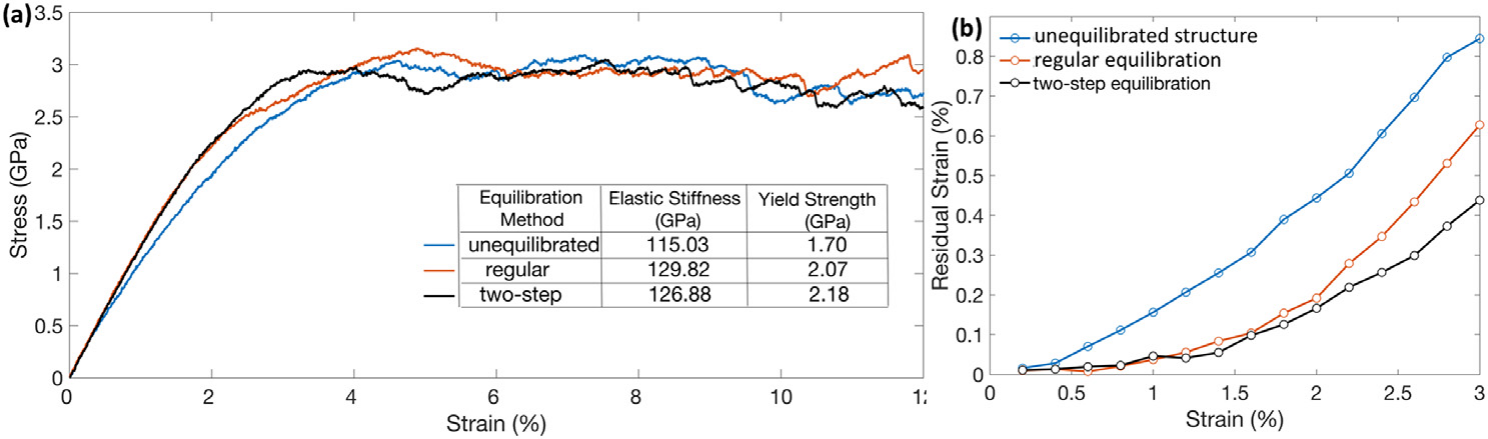
(a) Stress-strain curves obtained from the uniaxial testing and (b) residual strain curves obtained from the unloading simulations of the 6 nm grain size NC structure in different configurations: initial unequilibrated, equilibrated via the regular and the two-step equilibration methods.

Fig. 8(a) shows the stress-strain curve obtained from the uniaxial tensile testing of NC structure equilibrated using the two-step method. For comparison, it is plotted against the stress-strain curves of the initial unequilibrated structure and the structure obtained from the regular equilibration method. The initial elastic stiffness and the yield strength values are also shown. In terms of elastic and yielding behavior, both the two-step equilibrated, and the regular equilibrated NC structures are stiffer than the unequilibrated structure. However, the exact values of the stiffness and the yield strength, as obtained from different equilibration methods, are slightly different. This behavior is also reflected in their residual strain curves shown in Fig. 8(b). The residual strain in the properly equilibrated NC structures, either obtained from the two-step or the regular equilibration method, are similar in values till macroscopic yielding of the samples (1.5%–2% applied strain). The values diverge after yielding but always remain much lower than the residual strain values of the initial unequilibrated NC structure. Therefore, this comparison indicates the final equilibrated structures obtained from the two-step and the regular equilibration methods, are much closer to each other than the initial unequilibrated structure in terms of the microstructure statistics and the mechanical properties. Slight disparities in the resulting properties from these equilibration methods are expected since the total energy reductions are also not equal in the two cases.

### Concluding Remarks

The present work investigates the effect of thermal equilibration of atomistic NC structures on the resulting mechanical behavior. The results indicate that well-equilibrated NC structures are stiffer in terms of both elastic response and yielding behavior and accumulate less residual strain upon unloading, as compared with the otherwise unequilibrated NC structures. These outcomes highlight the significance of proper equilibration in atomistic simulations. However, it is found that the regular 300 K thermal equilibration method, typically employed in modeling studies, takes significantly longer for the system's energy to reach a local minimum, and thus a larger computational effort/expense. An improved two-step equilibration method is then proposed, for expediting the equilibration process in atomistic NC structures. This method ensured that the system reached some metastable equilibrium state rapidly without any significant grain growth. The effects of annealing time and temperature on the evolution of system's energy during the two-step equilibration are then studied and a set of optimal simulation parameters are established for Ni, as a starting point for other materials systems. Finally, it is shown that despite high temperature annealing conducted in the two-step method, the resulting microstructure has similar grain size as the one obtained after the regular equilibration method performed for significantly larger simulation times and comparable structural and mechanical properties.

## Declaration of Competing Interest

The authors declare that they have no known competing financial interests or personal relationships that could have appeared to influence the work reported in this paper.
